# Nitric Oxide Regulates Neurogenesis in the Hippocampus following Seizures

**DOI:** 10.1155/2015/451512

**Published:** 2015-10-26

**Authors:** Bruno P. Carreira, Daniela F. Santos, Ana I. Santos, Caetana M. Carvalho, Inês M. Araújo

**Affiliations:** ^1^Center for Neuroscience and Cell Biology, Neuroendocrinology and Neurogenesis Group, University of Coimbra, 3004-517 Coimbra, Portugal; ^2^Regenerative Medicine Program, Department of Biomedical Sciences and Medicine, University of Algarve, 8005-139 Faro, Portugal; ^3^Center for Biomedical Research (CBMR), University of Algarve, 8005-139 Faro, Portugal

## Abstract

Hippocampal neurogenesis is changed by brain injury. When neuroinflammation accompanies injury, activation of resident microglial cells promotes the release of inflammatory cytokines and reactive oxygen/nitrogen species like nitric oxide (NO). In these conditions, NO promotes proliferation of neural stem cells (NSC) in the hippocampus. However, little is known about the role of NO in the survival and differentiation of newborn cells in the injured dentate gyrus. Here we investigated the role of NO following seizures in the regulation of proliferation, migration, differentiation, and survival of NSC in the hippocampus using the kainic acid (KA) induced seizure mouse model. We show that NO increased the proliferation of NSC and the number of neuroblasts following seizures but was detrimental to the survival of newborn neurons. NO was also required for the maintenance of long-term neuroinflammation. Taken together, our data show that NO positively contributes to the initial stages of neurogenesis following seizures but compromises survival of newborn neurons.

## 1. Introduction

Neurogenesis, a multistep process that gives rise to functional and integrated new nerve cells from self-renewal and multipotent neural stem cells (NSC) [1, 2], occurs throughout adulthood in many animal species, including humans [3, 4]. Adult neurogenesis involves proliferation, migration, differentiation and fate determination, survival, maturation, and integration of newborn cells into the preexisting neuronal network. Two main areas are recognized as neurogenic niches in the adult mammalian brain: the subventricular zone (SVZ) of the lateral ventricles and the subgranular zone (SGZ) of the dentate gyrus of hippocampus. Particularly in dentate gyrus, new nerve cells are formed locally at the border between the granular layer and the hilus (SGZ), migrate short distances along the inner granular zone (IGZ), and extend long axonal projections to the CA3 pyramidal cell layer of the hippocampus [5, 6]. It has been shown that neurogenesis can be modulated by different physiological and environmental factors. Hormones, some growth factors, learning, exercise, and antidepressants seem to activate and stimulate the proliferation of NSC [7–9], while aging or inflammation has the opposite effect [10–12]. The neurogenic response to lesion involves neuroinflammation that activates resident microglia cells. In these conditions, microglia cells release inflammatory cytokines and reactive oxygen and nitrogen species like NO [13].

NO is a free radical gaseous molecule that results from the conversion of L-arginine into L-citrulline, catalyzed by the nitric oxide synthase family of enzymes. Particularly in inflammatory conditions, the expression of the inducible nitric oxide synthase (iNOS) is involved in the production of high levels of NO. NO is an important cellular messenger with different cell targets, being involved in many physiological mechanisms in cardiovascular, immunological, and nervous systems [14]. During neurogenesis, particularly in the early stages, such as proliferation, the neurogenic response mediated by NO depends on the pathophysiological state of the tissue, source of NO, and time of exposure [15]. Despite the intensive investigation on the effect of NO on the proliferation of NSC, there is a lack of information about the role of NO in migration, differentiation, and survival on newborn cells following brain injury.

In this work we investigated the role of NO from inflammatory origin in the regulation of hippocampal neurogenesis after a brain insult. We analyzed the proliferation of NSC, migration, differentiation, and survival of newborn cells using a KA-induced seizure mouse model [16, 17]. We show that NO increased the proliferation of early-born cells, particularly in the SGZ, and the number of neuroblasts, following* status epilepticus* (SE). Furthermore, NO was important for the maintenance of long-term neuroinflammation, which may be the leading cause to its detrimental effect on the survival of newborn cells in the dentate gyrus. Taken together, our data show that NO is a promising target for promoting the proliferation and migration of NSC following seizures, although its presence may compromises long-term survival of newborn cells.

## 2. Materials and Methods

### 2.1. Materials

5-Bromo-2′-deoxyuridine (BrdU), normal goat serum (NGS), paraformaldehyde (PFA), and Triton X-100 were purchased from Sigma Chemical (St. Louis, MO, USA). Mouse anti-neuronal nuclear (NeuN) and mouse anti-glial fibrillary acid protein (GFAP) were purchased from Millipore (Billerica, MA). DAKO fluorescent mounting medium was obtained from DakoCytomation (Glostrup, Denmark). Rat anti-BrdU was obtained from Oxford Biotechnology and doublecortin (C-18) (DCX) from Santa Cruz Biotechnology (Dallas, Texas, USA). Rabbit anti-cleaved caspase-3 was obtained from Cell Signaling (Danvers, MA, USA). Anti-rat and anti-rabbit IgG conjugated with Alexa Fluor 488 and anti-rat and anti-mouse IgG conjugated with Alexa Fluor 594 secondary antibodies were purchased from Molecular Probes (Invitrogen, Paisley, UK). Kainic acid was obtained from Ocean Produce (Canada) and sodium thiopental from B. Braun Melsungen (Germany).

### 2.2. Animals

Two-month-old C57BL/6J (iNOS^+/+^) mice or B6.129P2-*Nos2* ^*tm1Lau*^/J (iNOS^−/−^) male mice were obtained from Charles River (Barcelona, Spain) or The Jackson Laboratory (Bar Harbor, ME, USA), respectively. The animals were kept in animal facilities with food and water* ad libitum* in a 12 : 12 light-dark cycle. The weight of the animals varied between 18 and 26 g. All experiments were performed in accordance with NIH and European guidelines (86/609/EEC) for the care and use of laboratory animals. Furthermore, the animals were housed in our licensed animal facility (International Animal Welfare Assurance number 520.000.000.2006). In addition, all the people working with animals have received appropriate education (FELASA course) as required by the Portuguese authorities. This study is included in two projects approved and financed by the Foundation for Science and Technology (FCT, Portugal, PTDC/SAU-NEU/102612/2008 and PTDC/NEU-OSD/0473/2012) that approved the animal experimentation described. The animal experimentation board at the Center for Neurosciences and Cell Biology also approved the use of the animals in this study.

### 2.3. Administration of Kainic Acid in Mice

KA was dissolved in a sterile saline solution (0.9% NaCl in water) and injected subcutaneously (25 mg/kg), as previously described by our group [15]. All animals that received KA developed grade five seizures or higher according to 1972s Racine's six-point scale modified for mice [18]. In animals injected with saline solution alone, no seizures were observed and were used as controls. At least three animals survived in each experimental group.

### 2.4. Assessment of NSC Proliferation by 5-Bromo-2′-deoxyuridine Incorporation

**Figure 1:**
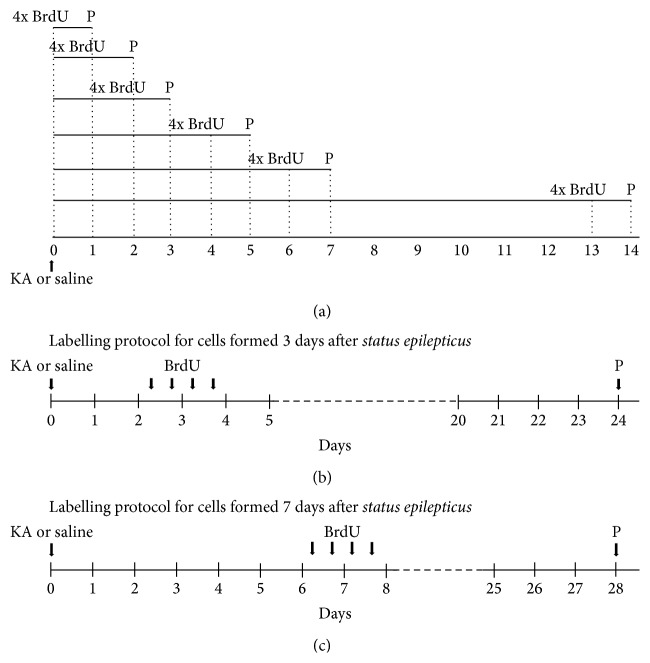
Experimental protocol for assessment of NSC proliferation (a) and differentiation (b and c). (a) KA or saline solutions were subcutaneously injected (25 mg/kg). Intraperitoneal injections (i.p.) of BrdU (4 doses, 50 mg/kg) were administrated every 2 hours, up to 12 hours before transcardiac perfusion (P). (b) Administration protocol of BrdU 3 days after SE. (c) Administration protocol of BrdU 7 days after SE. Intraperitoneal injections (i.p.) of BrdU (4 doses, 50 mg/kg) were administrated every 12 hours. Perfusions (P) were performed 3 weeks after BrdU treatment, following anesthesia.

To assess proliferation of NSC, all animals treated with KA or saline solution were treated with BrdU (intraperitoneal (i.p.) injections, 4 doses, 50 mg/kg) 2 hours apart, in a total of 200 mg/kg, up to 12 hours before sacrificing the animals at different time points (Figure 1(a)). In order to analyze distribution of NSC along dentate gyrus, all animals were treated with BrdU (i.p. injections, 4 doses, 50 mg/kg) every 12 hours, three and seven days after KA or saline administration. Three weeks later, mice were sacrificed (Figures 1(b) and 1(c)). In both experiments mice were transcardially perfused with 0.9% NaCl followed by 4% PFA in 0.01 M phosphate buffer saline (PBS, 7.8 mM Na_2_HPO_4_·2H_2_O, 2.7 mM NaH_2_PO_4_·H_2_O, 154 mM NaCl, and pH 7.2), following deep anesthesia with Eutasil (20% sodium pentobarbital). Brains were removed and kept overnight in 4% PFA for further fixation and then dehydrated in 20% sucrose/0.2 M phosphate buffer (PB, 48 mM NaH_2_PO_4_·H_2_O, 152 mM Na_2_HPO_4_·2H_2_O, and pH 7.2), at 4°C. Coronal sections from the hippocampal region were cryosectioned (30 *μ*m thick, in 8 series) and stored in an antifreeze solution (0.05 M PB, 30% ethylene glycol, and 30% glycerol), at 4°C.

### 2.5. Immunohistochemistry

Free-floating coronal hippocampal sections were processed for immunohistochemistry against DCX or BrdU and NeuN, BrdU, and GFAP or BrdU and cleaved caspase-3. Brain sections were treated with 1 M HCl for 20 min at 65°C, for DNA denaturation and then blocked for 1 hour with 5% NGS in 0.25% Triton X-100 in 0.01 PBS. Slices were then incubated with the primary antibodies, goat anti-DCX (1 : 400) or rat anti-BrdU (1 : 50) and mouse anti-NeuN (1 : 200) or mouse anti-GFAP (1 : 7000) or rabbit anti-cleaved caspase-3 (1 : 600), 48 h at 4°C. After rinsing with 0.25% Triton X-100 in PBS and with 2% block solution (NGS), the sections were incubated with the correspondent secondary antibodies (1 : 200), in 2% block solution (NGS), for 2 h in the dark, at room temperature. After rinsing with 0.25% Triton X-100 in PBS, the sections were kept in PBS 0.1 M solution, at 4°C, until setting in 2% gelatin-coated slides with DAKO fluorescence mounting medium.

### 2.6. Analysis of BrdU Incorporation

**Figure 2:**
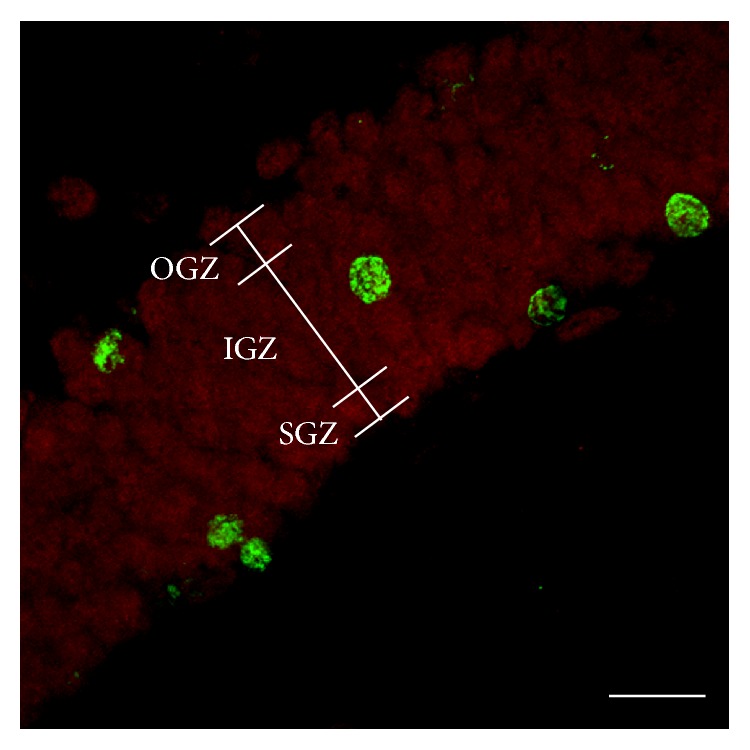
Schematic representation of the three zones of the dentate gyrus: the subgranular zone (SGZ), inner granular zone (IGZ), and outer granular zone (OGZ). The number of BrdU+ cells (green) was counted in each zone.

The distribution of the newborn cells in the dentate gyrus was analyzed in SGZ, IGZ and outer granular zone (OGZ) (Figure 2) and the numbers of BrdU+ cells were counted in each zone using an epifluorescence microscope (20x objective, Axiovert 200, Zeiss, Jena, Germany).

Images (0.73 *μ*m *z*-stacks) from 50 BrdU+ cells of each brain were acquired in a laser scanning microscope LSM 510 META or LSM 710 (Zeiss, Jena, Germany) with Argon/2 (488 nm) and DPSS 561-10 (561 nm) lasers (63x oil-immersion objective). Orthogonal projections in *y*-axis were performed and counted the number of BrdU+/NeuN+ or BrdU+/GFAP+ or BrdU+/Casp3+ cells were counted. The percentage of cells which show colocalization of markers was obtained by dividing the total number of BrdU+/NeuN+ or BrdU+/GFAP+ or BrdU+/Casp3+ cells by 50 BrdU+ cells.

### 2.7. DCX and GFAP Immunoreactivity

DCX and GFAP immunoreactive areas were analyzed using ImageJ software. Snap images were acquired in a Zeiss Axioimager (Zeiss, Jena, Germany) with a 20x objective. The threshold value was set for each staining and the percentage of dark background area was measured, excluding more anterior and posterior dentate gyri.

### 2.8. Statistical Analysis

The data are expressed as means ± SEM. Statistical significance was determined by using two-factor analysis of variance (ANOVA), followed by* post hoc* Bonferroni's test in GraphPad Prism 5 software. Differences were considered significant when *p* < 0.05.

## 3. Results and Discussion

### 3.1. NO Is Involved in the Proliferation of NSC in the Dentate Gyrus following SE

#### 3.1.1. Proliferation of Neural Stem Cells in the Hippocampus following Seizures Comprises a NO-Dependent and NO-Independent Phase

**Figure 3:**
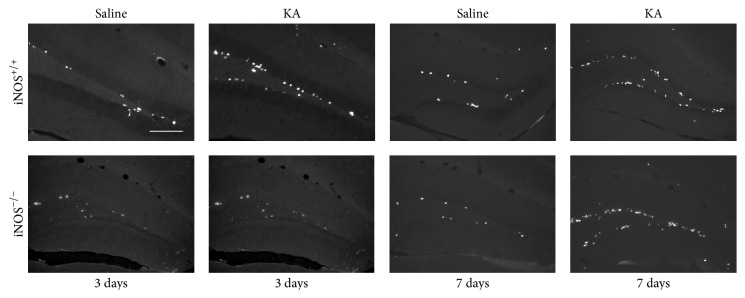
Number of BrdU+ cells in the dentate gyrus increased with KA treatment. Representative images of BrdU+ cells (white), 3 and 7 days after KA or saline treatment in iNOS^+/+^ or iNOS^−/−^ mice. Scale bar: 50 *μ*m.

In order to investigate the role of NO in cell proliferation, we used an* in vivo* KA-induced seizure mouse model, as described in Section 2. Proliferation of newborn cells was evaluated by the incorporation of BrdU, a thymidine analogue. The number of BrdU+ cells in the dentate gyrus was assessed by immunohistochemistry (Figure 3).

In iNOS^+/+^ mice, treatment with KA increased significantly the incorporation of BrdU in the SGZ from 3 days after treatments up to 14 days, when compared to saline-treated mice (Table 1, two-factor ANOVA; treatment: 31.95, *F* = 151.5, *df* = 3; time: 29.71, *F* = 84.57, *df* = 5; treatment × time (interaction): 33.70, *F* = 31.97, *df* = 15), with a peak at 5 days after treatment with KA (82.70 ± 5.87 cells/section, *p* < 0.001). In iNOS^+/+^ mice treated with saline solution, the number of BrdU+ cells did not change significantly during the analyzed period of time (*p* > 0.05 for all time points). These results are in line with previous findings that seizures in mice trigger neuroinflammation and stimulate cell proliferation in the SGZ of the dentate gyrus [11, 17, 19, 20]. Cell proliferation in the hippocampus is also increased in different acute injured-animal models, such as stroke [21, 22] and traumatic brain injury [23, 24].

In iNOS^−/−^ mice, BrdU incorporation was unchanged with KA treatment up to 5 days after seizures (7.73 ± 1.43 cells/section at 1 day after SE (*p* > 0.05), 13.35 ± 3.87 cells/section for 2 days after seizures (*p* > 0.05), 25.64 ± 0.53 cells/section for 3 days after SE (*p* > 0.05), and 18.36 ± 1.99 cells/section for 5 days after SE (*p* > 0.05)). Interestingly, the number of BrdU+ cells in the dentate gyrus of iNOS^−/−^ mice was significantly increased 7 days after seizures (87.08 ± 8.40 cells/section, *p* < 0.001), compared with saline-treated iNOS^−/−^ mice (20.08 ± 0.61 cells/section). Finally, the incorporation of BrdU returned to basal levels 14 days after seizures with KA treatment (27.33 ± 2.17 cells/section, *p* > 0.05). In saline-treated iNOS^−/−^ mice the incorporation of BrdU was similar for all time points (*p* > 0.05). In these animals, proliferation seems to be regulated by two different mechanisms: one that is NO-dependent up to 5 days after seizures and another that is regulated by a NO-independent mechanism, at 7 days after seizures.

Production of inflammatory factors from microglia, such as NO, has already been reported as essential for proliferation of NSC in the hippocampus [25]. Our group previously described the mechanism by which NO triggers the initial proliferation in SVZ cells [15, 26]. In those studies, we reported that NO is able to bypass the epidermal growth factor receptor and directly activate upstream components of ERK 1/2/MAPK signaling pathway, resulting in increased cell proliferation of NSC in early stages [15].

Moreover, late proliferation depends on the activation of cGMP and PKG, suggesting a biphasic mechanism of proliferation trigged by NO [9]. Interestingly, we observed an increase in proliferation of NSC 7 days after seizures in iNOS^−/−^ mice, which suggests that proliferation at this time is independent of NO. There are some other potential signaling pathways that may play a role in NSC proliferation at this time point. For instance, the NO-cGMP pathway is an important mediator of the proliferative effects of neuropeptide Y in the hippocampus [27–29]. Also in a model of SE, activated microglia induce the expression of insulin-like growth factor-1 (IGF-1) and stimulates the proliferation of progenitor cells in SGZ by a MAPK-dependent mechanism [30]. There are several factors expressed by microglia cells that can regulate neurogenesis and NSC can also regulate the activation of microglia cells, so it is possible that this interaction microglia-NSC may function as some kind of compensatory mechanism to regulate proliferation of NSC, independently of NO.

### 3.2. Involvement of NO on Migration and Distribution of Newborn Cells in the Dentate Gyrus following Seizures

#### 3.2.1. Distribution of Newborn Cells Formed 3 Days after Seizures in the Dentate Gyrus Is Independent of NO

**Figure 4:**
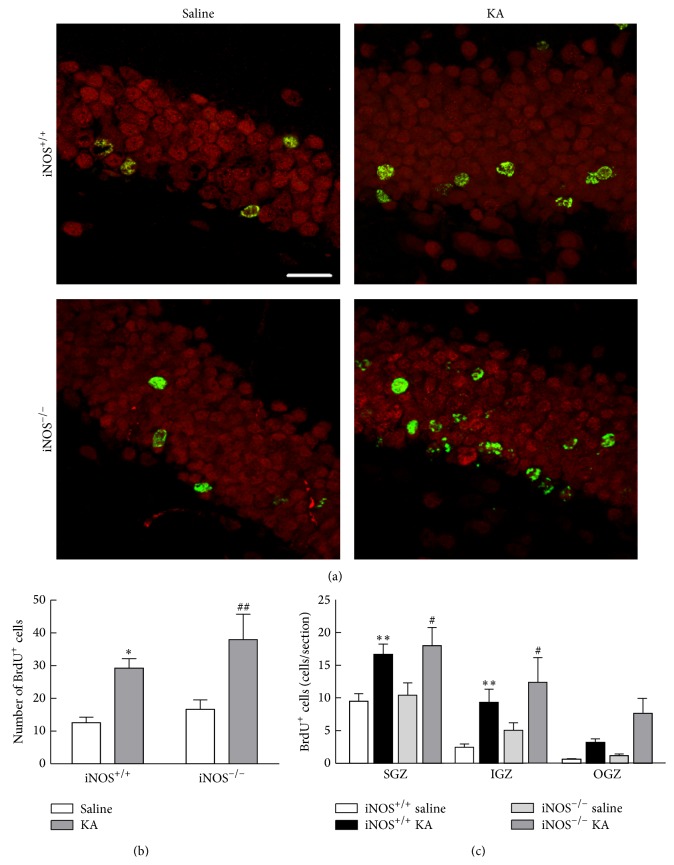
The number of BrdU+ cells in the dentate gyrus increased following seizures, 21 days after BrdU treatment, by a NO-independent mechanism. (a) Representative images of the distribution of BrdU (green) and NeuN (red) positive cells in different layers of the dentate gyrus of the hippocampus, 3 days after induction of seizures with KA or saline treatment, in iNOS^+/+^ and iNOS^−/−^ mice. Scale bar: 20 *μ*m. (b) Number of BrdU+ cells in iNOS^+/+^ and iNOS^−/−^ mice. Data are expressed as means ± SEM. Two-factor ANOVA (Bonferroni's posttest): *N* = 5 to 7; ^*∗*^ *p* < 0.05, significantly different from iNOS^+/+^ saline; ^##^ *p* < 0.01, significantly different from iNOS^−/−^ saline. (c) BrdU+ cells in different layers of the dentate gyrus, namely, in the SGZ, IGZ and OGZ of iNOS^+/+^ vs iNOS^−/−^ mice, 3 days following SE. Data are expressed as means ± SEM. Two-factor ANOVA (Bonferroni's posttest): *N* = 5 to 7; ^*∗∗*^ *p* < 0.01, significantly different from iNOS^+/+^ saline; ^#^ *p* < 0.05, significantly different from iNOS^−/−^ saline.

We next investigated the role of NO in the distribution of newborn cells in the dentate gyrus after 21 days following proliferation dependent on NO (that occurs at 3 days post-seizure), to evaluate whether the cells remained in the subgranular zone or are redistributed to the outer layers of the dentate gyrus. iNOS^+/+^ or iNOS^−/−^ mice were treated with either saline or KA and BrdU was injected in all animals 3 days later. The distribution of the new cells formed at this time point was assessed in the SGZ, IGZ, and OGZ of the dentate gyrus, 21 days after BrdU administration. BrdU+ cells increased with KA treatment in iNOS^+/+^ and iNOS^−/−^ mice (Figure 4(a)). The total number of BrdU+ cells significantly increased with KA treatment in both iNOS^+/+^ and iNOS^−/−^ mice (Figure 4(b), two-factor ANOVA; genotype: 5.68, *F* = 2.334, *df* = 1, *p* > 0.05; treatment: 49.79, *F* = 20.47, *df* = 1, *p* < 0.001; genotype × treatment (interaction): 0.76, *F* = 0.3113, *df* = 1, *p* > 0.05). For iNOS^+/+^ mice, treatment with KA duplicated BrdU+ cells (29.24 ± 2.91 BrdU+ cells/section, *p* < 0.05) compared to saline-treated mice (12.59 ± 1.66 BrdU+ cells/section). For iNOS^−/−^ mice, KA treatment also doubled the number of BrdU+ cells (37.99 ± 7.75 BrdU+ cells/section, *p* < 0.01) compared to saline-treatment (16.66 ± 2.89 BrdU+ cells/section).

In iNOS^+/+^ mice, KA treatment significantly increased BrdU+ cells in SGZ (16.68 ± 1.56 BrdU+ cells/section, *p* < 0.01) comparatively to saline-treated mice (9.49 ± 1.18 BrdU+ cells/section) (Figure 4(c), two-factor ANOVA; treatment: 25.52, *F* = 14.81, *df* = 3, and *p* < 0.001; regions: 41.85, *F* = 36.43, *df* = 2, and *p* < 0.001; treatment × regions (interaction): 1.62, *F* = 0.4690, *df* = 6, and *p* > 0.05). For these mice, BrdU+ cells also increased with KA treatment in IGZ (9.35 ± 1.97 BrdU+ cells/section, *p* < 0.01) when compared with saline-treated mice (2.48 ± 0.47 BrdU+ cells/section). BrdU+ cells did not change significantly in OGZ with KA treatment. Similarly, in iNOS^−/−^ mice (Figure 4(c)), BrdU+ cells significantly increased after seizures in SGZ (17.98 ± 2.82 BrdU+ cells/section, *p* < 0.05) and IGZ (12.37 ± 3.78 BrdU+ cells/section, *p* < 0.05), compared with saline-treated iNOS^−/−^ mice. These results suggest that the distribution of NSC born 3 days after the insult is regulated by a NO-independent mechanism.

#### 3.2.2. Abolishment of NO Does Not Affect Distribution of Newborn Cells Formed 21 Days following Seizures in the Dentate Gyrus

We next investigated the role of NO in the distribution of newborn cells in the dentate gyrus after 21 days following proliferation during a phase that is not dependent on NO (that occurs at 7 days post-seizure). iNOS^+/+^ or iNOS^−/−^ mice were treated with either saline or KA, BrdU was injected in all animals 7 days later, and perfusions performed 28 days after BrdU injection (7 days after seizures followed by 21 days).

**Figure 5:**
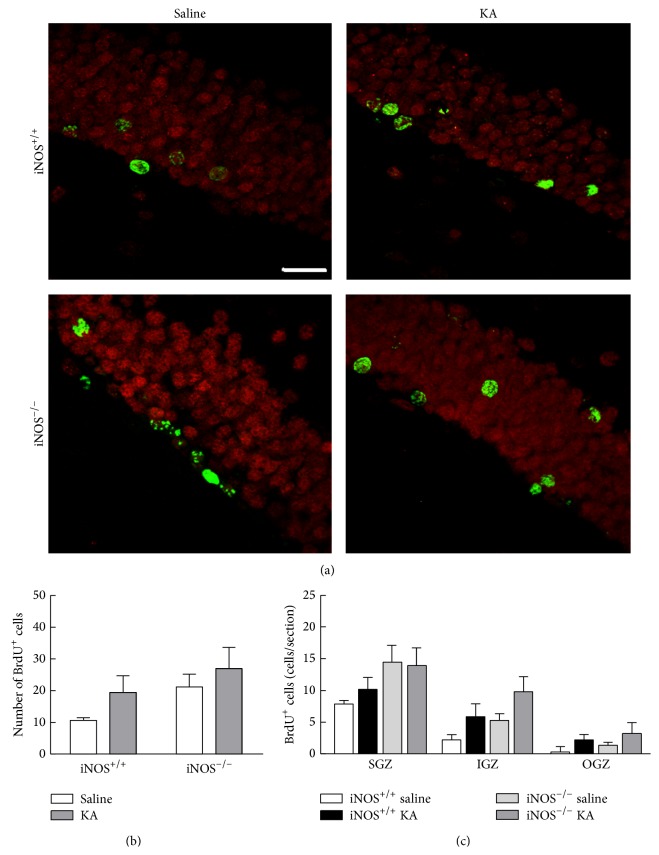
The number of BrdU+ cells in the dentate gyrus born 7 days after SE is not affected by NO, 21 days after treatment with BrdU. (a) Representative images of BrdU (green) and NeuN (red) positive cells in the dentate gyrus, 7 days after treatment with KA or saline in iNOS^+/+^ and iNOS^−/−^ mice. Scale bar: 20 *μ*m. (b) Number of BrdU+ cells in iNOS^+/+^ and iNOS^−/−^ mice. Data are expressed as means ± SEM. Two-factor ANOVA (Bonferroni's posttest), *N* = 4 to 6, *p* > 0.05. (c) BrdU+ cells in the three regions of the dentate gyrus. Data are expressed as means ± SEM. Two-factor ANOVA (Bonferroni's posttest), *N* = 4 to 6, *p* > 0.05.

Treatment with KA in cells formed 7 days after seizures did not change significantly the number of BrdU+ cells along the dentate gyrus (21 days after the cells were labeled with BrdU around day 7) for none of the genotypes (Figures 5(a) and 5(b), two-factor ANOVA, treatment: 10.61, *F* = 2.180, *df* = 1, and *p* > 0.05; genotype: 15.95, *F* = 3.278, *df* = 1, and *p* > 0.05; treatment × genotype (interaction): 0.45, *F* = 0.09155, *df* = 1, and *p* > 0.05). In iNOS^+/+^ and iNOS^−/−^ mice, the number of BrdU+ cells in KA-treated mice was similar in all zones of the dentate gyrus, compared to the respective saline controls (Figure 5(c), two-factor ANOVA; treatment: 11.25, *F* = 4.592, *df* = 3, and *p* < 0.01; regions: 48.07, *F* = 29.44, *df* = 2, and *p* < 0.001; treatment × regions (interaction): 3.95, *F* = 0.8072, *df* = 6, and *p* > 0.05).

According to our study, at 7 days after KA treatment the proliferation of newborn cell is regulated by a NO-independent mechanism. As described before, there are some other factors that can lead to activation of signaling pathways possible involved in regulation of proliferation of NSC in the hippocampus at this stage of the neurogenesis. For instance, IGF-1 produced by activated microglia cells can increase the proliferation of NSC in the SGZ by a mechanism dependent of the MAPK signaling [30]. Also there is the possibility of a compensatory mechanism regulated by NSC, independently of any other factor produced by microglia cells. Thus, the distribution of newborn cells in the dentate gyrus was similar in iNOS^+/+^ and iNOS^−/−^ mice after seizures, suggesting that NO is not involved in how cells are distributed along the dentate gyrus 7 days after seizures, but other factors may be involved.

### 3.3. NO Has Different Effects in Neuronal and Astrocytic Differentiation

#### 3.3.1. Early Neuronal Differentiation following Seizures Is Dependent on NO

Here, we identified immature neurons in iNOS^+/+^ and iNOS^−/−^ mice treated with either saline or KA, as described in Section 2. DCX is a specific marker for neuroblasts and immature neurons [31] born in the first two weeks of the neurogenic process. Accordingly, we chose to analyze DCX-immunoreactive area at 7 days and 14 days after KA treatment.

**Figure 6:**
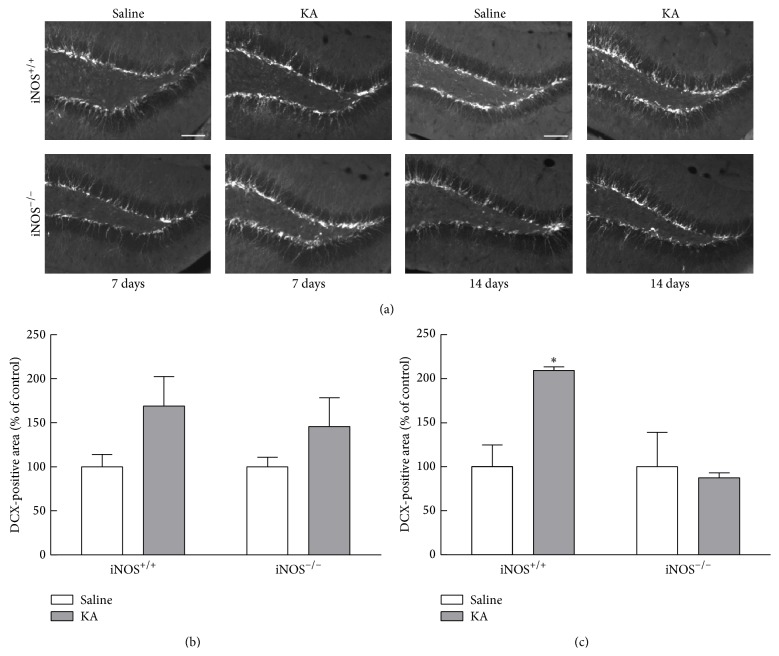
DCX immunoreactivity is dependent on NO, 14 days after seizures. (a) Representative images of DCX (white) immunoreactivity in the dentate gyrus, 7 and 14 days after KA or saline treatment in iNOS^+/+^ and iNOS^−/−^ mice. Scale bar: 100 *μ*m. (b) DCX-immunoreactive area 7 days after SE. (c) DCX-immunoreactive area 14 days after SE. Data are expressed as means ± SEM. Two-factor ANOVA (Bonferroni's posttest): *N* = 3 to 6, ^*∗*^ *p* < 0.05, significantly different from iNOS^+/+^ saline.

The DCX-immunoreactive area was increased in iNOS^+/+^ mice, 14 days after seizures compared to saline-treated mice, but not in KA-treated iNOS^−/−^ compared to saline-treated mice (Figure 6(a)). At 7 days after seizures, the percentage of DCX-immunoreactive area tends to increase with KA treatment in both iNOS^+/+^ (169.01 ± 33.50% of control, *p* > 0.05) and iNOS^−/−^ (145.64 ± 32.75% of control, *p* > 0.05), although this increase is not significant compared to saline-treated mice (Figure 6(b), two-factor ANOVA; genotype: 0.79, *F* = 0.1382, *df* = 1, and *p* > 0.05; treatment: 18.90, *F* = 3.326, *df* = 1, and *p* > 0.05; genotype × treatment (interaction): 0.78, *F* = 0.1381, *df* = 1, and *p* > 0.05). Later, at 14 days after seizures, the DCX+ area doubled in iNOS^+/+^ mice (209.32 ± 4.07% of control, *p* < 0.05), when compared to saline-treated mice of the same genotype (100.00 ± 24.75% of control). In iNOS^−/−^ mice, treatment with KA did not change the DCX-immunoreactive area (87.57 ± 5.48% of control, *p* > 0.05), at 14 days after seizures, when compared with the saline-treated mice (100.00 ± 39.13). (Figure 6(c), two-factor ANOVA; genotype: 23.01, *F* = 6.420, *df* = 1, and *p* < 0.05; treatment: 14.57, *F* = 4.065, *df* = 1, and *p* > 0.05; genotype × treatment (interaction): 23.01, *F* = 6.420, *df* = 1, and *p* < 0.05).

Our results showed that NO from an inflammatory origin increases the number of neuroblasts/immature neurons in the dentate gyrus, at least at 2 weeks after seizures. It has been shown that the number of DCX+ neuroblasts significantly increased following treatment with L-NAME, a NOS inhibitor, and KA together [32]. Moreover, in the same study, inhibition of NOS alone increased the number of BrdU+ newborn cells in the hilus, which suggests a role for NO in their correct migration into the granular zone of the dentate gyrus.

#### 3.3.2. NO Limits Survival of the Cells That Proliferate Earlier (3 Days) but Not Later (7 Days) after Seizures

**Figure 7:**
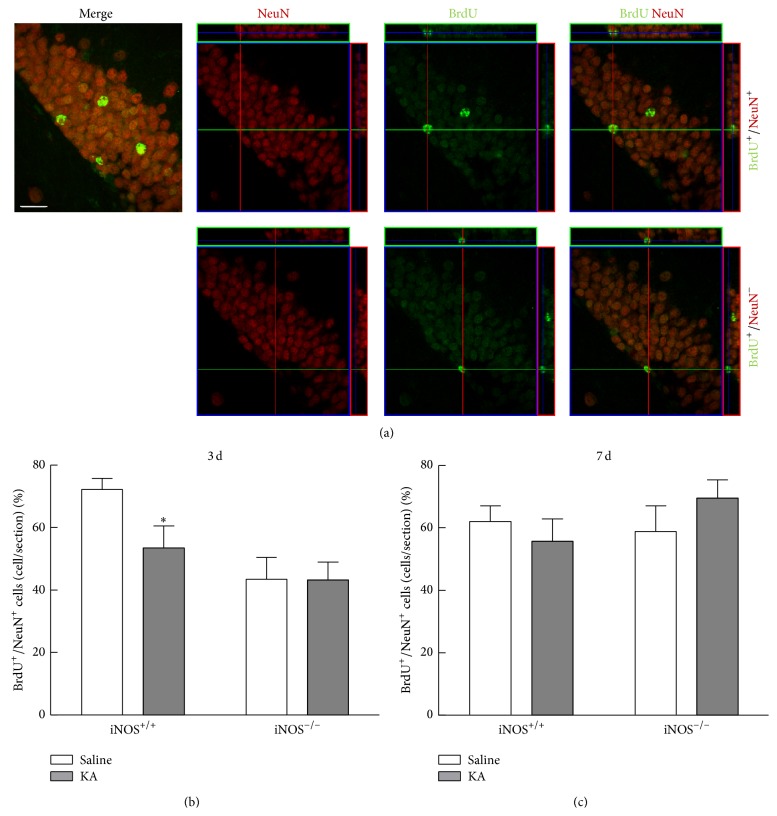
NO decreases the number of newborn neurons born 3 days after seizures in iNOS^+/+^ mice. (a) Orthogonal projections of representative images of BrdU-positive cells (BrdU+) shown green and NeuN-positive (NeuN+) cells, shown red. Scale bar: 20 *μ*m. Assessment of the percentage of BrdU-NeuN colocalizing cells in the dentate gyrus of iNOS^+/+^ or iNOS^−/−^ mice, 3 days (b) or 7 days (c) following SE. At least 3 surviving animals were used for each experimental group. Data are expressed as means ± SEM. Two-factor ANOVA (Bonferroni's posttest): *N* = 4 to 7; *p* < 0.05, significantly different from iNOS^+/+^ saline.

To investigate the survival at 21 days of cells formed 3 and 7 days after seizures, colocalization of BrdU+/NeuN+-cells was assessed by immunohistochemistry. NeuN is a neuronal marker for mature neurons and colocalization with BrdU allows the investigation of new neurons formed at the time point of treatment with BrdU. Images of 50 BrdU+ cells of each animal were acquired and orthogonal projections in *y*-axis were performed for each image (Figure 7(a)).

At 21 days after treatment with BrdU, the percentage of new neurons born 3 days after seizures decreased in iNOS^+/+^ mice treated with KA (53.50 ± 7.04% of BrdU+/NeuN+-cells, *p* < 0.05), compared to saline-treated mice (72.29 ± 3.48% of BrdU+/NeuN+-cells), but not in iNOS^−/−^ mice (43.50 ± 6.95% of BrdU+/NeuN+-cells for saline and 43.20 ± 5.68% of BrdU+/NeuN+-cells for KA-treated mice, *p* > 0.05) (Figure 7(b), two-factor ANOVA: genotype: 7.79, *F* = 2.682, *df* = 1, and *p* > 0.05; treatment: 32.65, *F* = 11.25, *df* = 1, and *p* < 0.01; genotype × treatment (interaction): 7.30, *F* = 2.516, *df* = 1, and *p* > 0.05). These results suggest that survival of newborn cells after seizures is regulated by a NO-dependent mechanism, similar to proliferation of NSC in these conditions. However, NO seems to result in the formation of less new neurons after seizures.

NO may be toxic to neurons and neuronal apoptosis was evident after administration of a NO donor in a febrile seizure rat model [33]. NO has also been proposed as an inhibitor of cell-cycle progression in many cell types, through activation of p53 or Rb signaling pathways [34, 35]. This relationship of NO and programmed cell death might influence the survival rate of the newborn cells. Moreover, NO does not necessarily need to be directly toxic to newborn neurons but may be involved in maintenance of inflammation that will condition the survival of the new cells.

For neurons born 7 days after seizures, the number of new neurons in iNOS^+/+^ mice was very similar between treatments, with 55.6 ± 7.22% of BrdU+/NeuN+-cells (*p* > 0.05) in KA-treated mice and 62.00 ± 5.03% of BrdU+/NeuN+-cells for saline-treated mice. In iNOS^−/−^ mice, treatment with KA (69.50 ± 5.85% of BrdU+/NeuN+-cells) also did not change the number of new neurons, compared to saline-treated mice (58.80 ± 8.31% of BrdU+/NeuN+-cells) (Figure 7(c), two-factor ANOVA; genotype: 3.15, *F* = 0.5348, *df* = 1, and *p* > 0.05; treatment: 0.53, *F* = 0.09010, *df* = 1, and *p* > 0.05; genotype × treatment (interaction): 8.07, *F* = 1.372, *df* = 1, and *p* < 0.05). These results suggest that cells that proliferate in a NO-independent phase become neurons that survive better than cells that proliferate earlier (3 days) after the onset of seizures.

**Figure 8:**
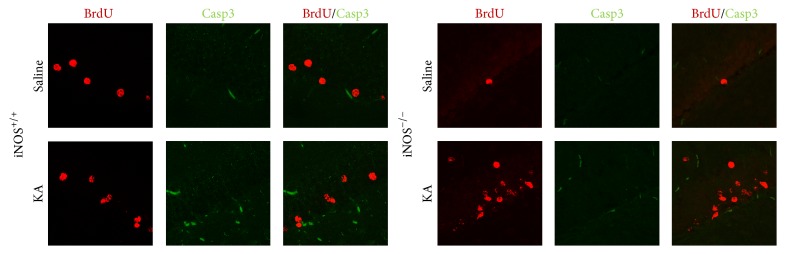
Absence of BrdU/Casp3 colocalization in cells formed 3 days after treatment with saline or KA in iNOS^+/+^ and iNOS^−/−^ animals. Representative images of BrdU-positive cells (red) and cleaved caspase-3 (Casp3, green). At least 3 surviving animals were used for each experimental group. Scale bar: 20 *μ*m.

In order to confirm the effect of NO in survival of the cells that proliferated 3 days after seizures, we assessed the colocalization of BrdU with a cell death marker, cleaved caspase-3, by immunohistochemistry. Representative images of each animal were acquired (Figure 8). Colocalization of BrdU with cleaved caspase-3 was not observed, which suggests that the decrease in the survival of the new neurons formed 3 days after seizures in iNOS^+/+^ animals does not result from apoptosis of the proliferating cells at this time point (21 days after treatment with BrdU). It is possible that cell death occurs earlier or by other mechanisms.

#### 3.3.3. Astrogliogenesis Is Not Affected by Abolishment of NO after Seizures

**Figure 9:**
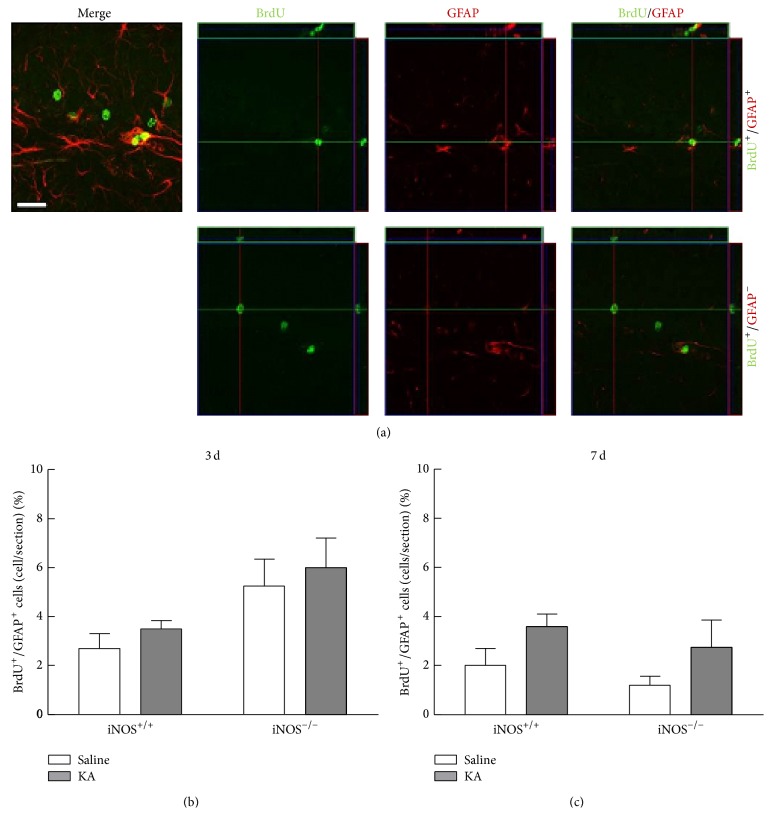
Differentiation of newborn cells formed 3 and 7 days after SE into astrocytes is not affected by NO. (a) Orthogonal projections of representative images of BrdU-GFAP colocalizing cells. Scale bar: 20 *μ*m. Percentage of BrdU-GFAP colocalizing cells, 3 days (b) or 7 days (c) following SE. Data are expressed as means ± SEM. Two-factor ANOVA (Bonferroni's posttest), *N* = 4 to 7, *p* > 0.05.

We were interested in understanding whether the proliferating cells could be differentiating into astrocytes. In order to analyze this, we assessed GFAP+ cells formed 3 and 7 days after seizures by immunohistochemistry 21 days after BrdU injection. GFAP is a protein expressed by astrocytes, and colocalization with BrdU allows the identification of newborn astrocytes at the time point of treatment with BrdU (Figure 9(a)).

In iNOS^+/+^ or in iNOS^−/−^ mice, seizures did not change the number of new astrocytes (BrdU-GFAP colocalizing cells) 3 days following SE (3.50 ± 0.34%, *p* > 0.05), compared with saline-treated mice (2.71 ± 0.61%) (Figure 9(b), two-factor ANOVA, genotype: 36.46, *F* = 10.30, *df* = 1, and *p* < 0.01; treatment: 3.39, *F* = 0.9584, *df* = 1, and *p* > 0.05; genotype × treatment (interaction): 0.00, *F* = 0.0005183, *df* = 1, and *p* > 0.05).

Furthermore, seizures did not change the number of BrdU-GFAP colocalizing cells in both genotypes, 7 days after SE (3.60 ± 0.51% or 2.75 ± 1.11% of BrdU-GFAP colocalizing cells in iNOS^+/+^ mice or iNOS^−/−^ mice, resp., *p* > 0.05). The percentage of BrdU-GFAP co-localizing cells in saline-treated mice was very similar between iNOS^+/+^ and iNOS^−/−^ mice (2.00 ± 0.71% versus 1.20 ± 0.37%, resp.) (Figure 9(c), two-factor ANOVA; genotype: 7.07, *F* = 1.473, *df* = 1, and *p* > 0.05; treatment: 25.76, *F* = 5.369, *df* = 1, and *p* < 0.05; genotype × treatment (interaction): 0.01, *F* = 0.001353, *df* = 1, and *p* > 0.05).

*In vitro* studies reported that exposure to pathological levels of NO (0.1 mM for 24 hours) promotes astroglial fate determination in NSC over neuronal commitment or selectively depletes early neuronal progenitor cells [36]. In this particular model, astrogliogenesis seems to be positively regulated by exposure to NO. Here we show that exposure to NO originated from iNOS is not involved in astroglial differentiation from neural stem cells after a brain injury, which did not change* per se* the number of newborn GFAP+ cells.

### 3.4. NO Is Important for Astrogliosis in iNOS^+/+^ Mice 28 Days after Treatment

**Figure 10:**
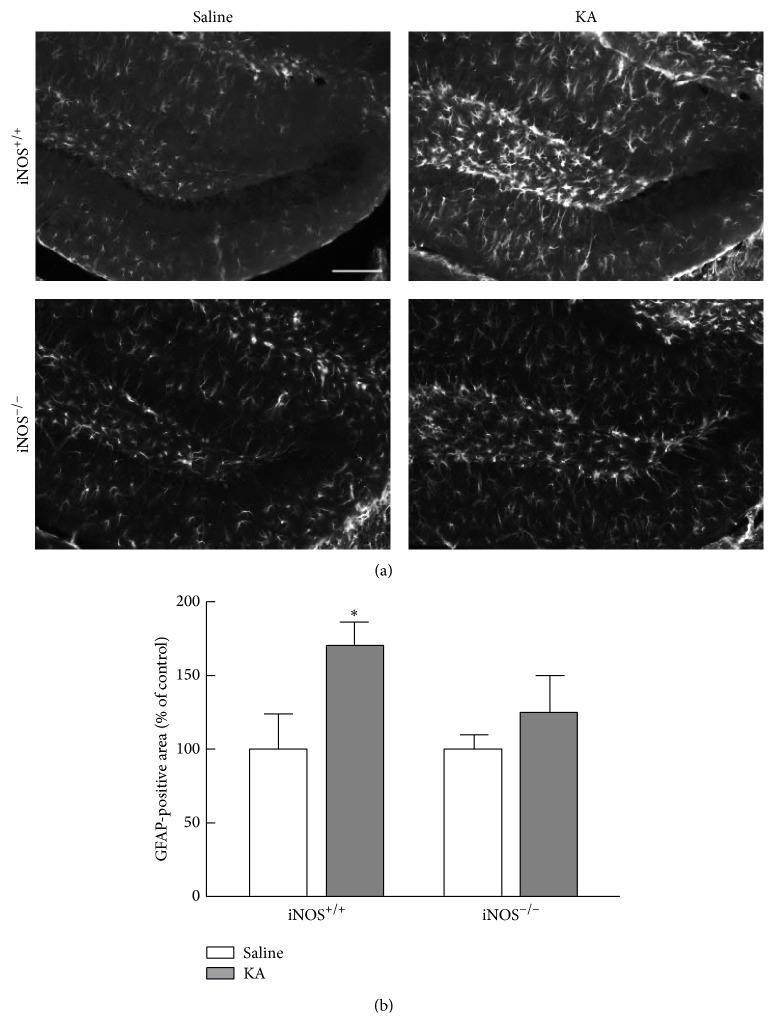
Astrogliosis is affected by abolishment of NO, 28 days after seizures. (a) Representative images of GFAP (white) immunoreactivity 28 days after KA or saline treatment in iNOS^+/+^ or iNOS^−/−^ mice. Scale bar: 100 *μ*m. (b) GFAP immunoreactivity 28 days following SE. Data are expressed as means ± SEM. Two-factor ANOVA (Bonferroni's posttest): *N* = 4 to 5; ^*∗*^ *p* < 0.05, significantly different from iNOS^+/+^ saline.

Next, we evaluated the possible involvement of NO in neuroinflammation, 28 days after seizures. For this purpose, GFAP immunoreactivity was assessed by immunohistochemistry and the intensity of GFAP staining used as a measure for astrogliosis (Figure 10(a)). In iNOS^+/+^ mice, KA treatment increased GFAP immunoreactivity (170.45 ± 15.74% of control, *p* < 0.05) when compared to saline-treated mice (100.00 ± 23.87% of control), 28 days after treatment (Figure 10(b), two-factor ANOVA, genotype: 28.27, *F* = 6.721, *df* = 1, and *p* < 0.05; treatment: 6.41, *F* = 1.527, *df* = 1, and *p* > 0.05; genotype × treatment (interaction): 6.42, *F* = 1.527, *df* = 1, and *p* < 0.05). Here we show that GFAP-immunoreactive area was increased 28 days after seizures, in a NO-dependent manner, suggesting that neuroinflammation is still present at this time.

Previously, our group studied neuroinflammation 5 days after seizures and showed an increase in the number of reactive astrocytes in either iNOS^+/+^ or iNOS^−/−^ treated with KA; thus the process is independent of NO production [15]. Here we show that activation of astrocytes is maintained up to 28 days after seizures. However, the astrogliosis is not observed at this time point in the mice lacking iNOS, suggesting that late astrogliosis, but not early astrogliosis, is NO-dependent. This prolonged neuroinflammation may condition survival of the newborn neurons in iNOS^+/+^ KA-treated mice, as observed.

### 3.5. Regulation of Physiological versus Pathophysiological Neurogenesis by NO

The role of NO in regulation of neurogenesis is still unclear. Overall, NO seems to negatively regulate neurogenesis in physiological conditions, while in pathophysiological situations it shows proneurogenic action. Several studies reported a decrease in proliferation of NSC [37–39] and survival of the newborn cells [36]. NO can also modulate differentiation of new precursors by increasing neuronal [38, 39] or astrocytic differentiation [36].

After a brain insult, NO has been reported as proneurogenic factor, since an increase in proliferation of NSC is reported in most of the injury-induced models, including stroke and seizures [22, 40]. Although differentiation is positively regulated by NO following brain insults [38], the survival of the newborn cells seems to be decreased by NO [41].

Our results, together with previous findings, suggest that not only is proliferation of NSC regulated by NO-dependent mechanisms following a lesion, but also differentiation and survival of the newborn neurons are regulated by the presence of NO following seizures. The fact that NO is important to maintain neuroinflammation up to 28 days after seizures may have influence on survival of newborn cells and may contribute to the failure of new neurons to efficiently survive in such conditions.

## 4. Conclusions

With this work we aimed to understand the involvement of NO produced from iNOS in hippocampal neurogenesis in a* status epilepticus* mouse model. Our results showed that production of NO in an inflammatory context increased proliferation of the early-born NSC following a brain insult. Early differentiation of neuroblasts and immature neurons increased following seizures by a NO-dependent mechanism. We also showed that the distribution of newborn cells along the dentate gyrus was modified by seizures, but NO was not involved in this phenomenon. Furthermore, survival of new neurons formed at an early stage (3 days after seizures) is decreased by NO. In fact, NO is showed to be important in maintenance of neuroinflammation up to 28 days after seizures, which may provide an aggressive environment for the newborn cells, which fail to survive.

Altogether, these findings help to better understand the involvement of NO produced by iNOS in different stages of adult neurogenesis following injury and open the possibility to explore new NO-based therapeutic approaches to brain repair after an insult, knowing when NO is proneurogenic and when it impairs survival of newborn neurons.

## Figures and Tables

**Table 1:** NO increased NSC proliferation following treatment with KA.

Days	iNOS^+/+^ saline		iNOS^+/+^ KA		iNOS^−/−^ saline		iNOS^−/−^ KA	
	Mean	SEM	Mean	SEM	Mean	SEM	Mean	SEM
1	16,82	0,91	9,39	1,04	11,31	2,86	7,72	1,43
2	23,13	1,31	33,23	1,86	12,10	1,33	13,35	3,87
3	24,45	1,77	63,36^*∗∗∗*^	1,36	16,71	4,05	25,64	0,53
5	26,32	1,12	82,69^*∗∗∗*^	5,87	15,58	0,96	18,36	1,99
7	21,60	0,49	82,45^*∗∗∗*^	3,44	20,08	0,61	87,08^###^	8,39
14	21,71	1,77	37,48^*∗∗*^	1,99	17,15	1,38	27,33	2,16

Evaluation of cell proliferation in the subgranular zone (SGZ) of iNOS^+/+^ versus iNOS^−/−^ mice, at several time points after SE, assessed by BrdU incorporation. Following seizure induction, there is a time-dependent increase in cell proliferation in iNOS^+/+^ animals, peaking at day 5. In iNOS^−/−^ mice, only 7 days after SE the number of BrdU+ cells significantly increases in the SGZ. At least 3 surviving animals were used for each experimental group. Data are expressed as means ± SEM. Two-factor ANOVA: *N* = 3 to 5, ^*∗∗*^ *p* < 0.01 and ^*∗∗∗*^ *p* < 0.001, significantly different from iNOS^+/+^saline; ^###^ *p* < 0.001, significantly different from iNOS^−/−^ saline.

## Abbreviations

BrdU: 5-Bromo-2′-deoxyuridine
GFAP: Glial fibrillary acid protein
IGZ: Inner granular zone
iNOS: Inducible nitric oxide synthase
KA: Kainic acid
NeuN: Neuronal nuclear
NGS: Normal goat serum
NO: Nitric oxide
NSC: Neural stem cells
OGZ: Outer granular zone
PFA: Paraformaldehyde
PBS: Phosphate-buffered saline
SGZ: Subgranular zone
SE: * Status epilepticus*
SVZ: Subventricular zone
DCX: Doublecortin.
